# Clinical and laboratory characteristics of clozapine-treated patients with schizophrenia referred to a national immunodeficiency clinic reveals a B-cell signature resembling common variable immunodeficiency (CVID)

**DOI:** 10.1136/jclinpath-2019-206235

**Published:** 2020-02-24

**Authors:** Mark James Ponsford, Rachael Steven, Kathyrn Bramhall, Mathew Burgess, Sonali Wijetilleka, Emily Carne, Frances McGuire, Colin R Price, Mo Moody, Soha Zouwail, Tayyeb Tahir, Daniel Farewell, Tariq El-Shanawany, Stephen R A Jolles

**Affiliations:** 1 Immunodeficiency Centre for Wales, University Hospital of Wales, Cardiff, UK; 2 Tenovus Institute, Division of Infection & Immunity, Cardiff University, Cardiff, UK; 3 Department of Medical Biochemistry, University Hospital of Wales, Cardiff, UK; 4 Department of Medical Biochemistry, Medical School, Alexandria University, Alexandria, Egypt; 5 Liaison Psychiatry, University Hospital of Wales, Cardiff, UK; 6 Division of Population Medicine, School of Medicine, Cardiff University, Cardiff, UK

**Keywords:** immunodeficiency, psychiatry, antibodies, diagnostics, flow cytometry

## Abstract

**Aims:**

An association between antibody deficiency and clozapine use in individuals with schizophrenia has recently been reported. We hypothesised that if clozapine-associated hypogammaglobulinaemia was clinically relevant this would manifest in referral patterns.

**Methods:**

Retrospective case note review of patients referred and assessed by Immunology Centre for Wales (ICW) between January 2005 and July 2018 with extraction of clinical and immunological features for individuals with diagnosis of schizophrenia-like illness.

**Results:**

1791 adult patients were assessed at ICW during this period; 23 patients had a psychiatric diagnosis of schizophrenia or schizoaffective disorder. Principal indications for referral were findings of low calculated globulin and immunoglobulins. Clozapine was the single most commonly prescribed antipsychotic (17/23), disproportionately increased relative to reported use in the general schizophrenia population (OR 6.48, 95% CI: 1.79 to 23.5). Clozapine therapy was noted in 6/7 (86%) of patients subsequently requiring immunoglobulin replacement therapy (IgRT). Marked reduction of class-switched memory B cells (CSMB) and plasmablasts were observed in clozapine-treated individuals relative to healthy age-matched controls. Clozapine duration is associated with CSMB decline. One patient discontinued clozapine, with gradual recovery of IgG levels without use of IgRT.

**Conclusions:**

Our findings are consistent with enrichment of clozapine-treatment within schizophrenic individuals referred for ICW assessment over the last 13 years. These individuals displayed clinical patterns closely resembling the primary immunodeficiency common variable immunodeficiency, however appears reversible on drug cessation. This has diagnostic, monitoring and treatment implications for psychiatry and immunology teams and directs prospective studies to address causality and the wider implications for this patient group.

## Introduction

Schizophrenia is an enduring major psychiatric disorder affecting around 1% of the population.^1^ In addition to the debilitating psychiatric symptoms, it has major psychosocial consequences with an unemployment rate of 80%–90% and a life expectancy reduced by 10–20 years^1^ including suicide rates of approximately 5%.^2^ Societal costs in England alone are estimated to be £11·8 billion per year.^3^ Clozapine is a dibenzo-diazepine atypical antipsychotic and the only licensed medication, for approximately 30% of patients with treatment-resistant schizophrenia (TRS).^1 4^ There is increasing evidence linking clozapine with pneumonia-related admissions^5–7^ and mortality.^8–11^ Postulated mechanisms include sialorrhoea, sedation, agranulocytosis and aspiration. We recently reported an association between clozapine therapy in schizophrenia and hypogammaglobulinaemia,^12 13^ greater than that reported following rituximab and methotrexate therapy in rheumatoid arthritis.^14^ To better define the clinical and immunological abnormalities associated with clozapine use, we performed a retrospective case review of patients assessed at the Immunology Centre for Wales (ICW).

## Methods

Electronic case records for patients assessed at ICW between January 2005 and July 2018 to identify all individuals with a concomitant psychiatric diagnosis of schizophrenia or schizoaffective disorder. Indication for referral, medication and comorbidities, and immunological testing at initial assessment and treatments were extracted using a standardised proforma. Recurrent infection history was defined as ≥3 distinct antibiotic courses per year or serious infection requiring admission, as in the wider literature.^15^

All testing was performed in the United Kingdom Accreditation Service-accredited Medical Biochemistry & Immunology Laboratory at the University Hospital of Wales. Immunoglobulin levels (IgG, IgA and IgM) were assayed by nephelometry (Siemens BN2 Nephelometer; Siemens), serum electrophoresis (Sebia Capillarys 2; Sebia, Norcross, Georgia, USA) and, where appropriate, serum immunofixation performed (Sebia Hydrasys; Sebia). Antibody titres against *Haemophilus influenzae*and *Streptococcus pneumoniae* capsular polysaccharide and tetanus toxoid were determined by ELISA (The Binding Site, Birmingham, UK). Flow cytometry was performed using Beckman Coulter FC500 analyser. Lymphocyte phenotypes were analysed using Beckman Coulter Cyto-stat Tetrachrome reagents (CD45-FITC/CD4-RD1/CD8-ECD/CD3-PC5 and CD45-FITC/CD56-RD1/CD10-ECD/CD3-PC5), Flow-Count Fluorospheres and versalyse lysis solution. B-cell phenotyping was performed as previously described^16^ using the following antibodies: CD19-PE/Cy7 (Beckman Coulter), CD27-FITC (Serotec), CD21-PE (BD Pharmingen), CD38-FITC (Beckman Coulter), IgM Alexa-Flour 647 (Jackson ImmunoResearch), IgD-PE (Southern Biotech). Common variable immunodeficiency (CVID) and age-matched healthy controls were analysed as part of an anonymous sample exchange scheme run jointly with King’s College London. Reference ranges are provided within the text. Individual clinical, immune and treatment data are available in online supplementary file S1.

10.1136/jclinpath-2019-206235.supp1 Supplementary data

## Statistical analysis

Data were curated in Microsoft Excel. Fisher’s exact test and non-parametric Mann-Whitney U test, following D’Agostino and Pearson normality assessment, and curve fitting were conducted using GraphPad Prism V.6.07. Where immunoglobulin level was undetectable, the lower limit of detection (IgG 1.34 g/L; IgA 0.05 g/L and IgM 0.05 g/L) was used for data analysis, with density estimation and plotting performed in R (V.3.4.0). A two-tailed significance level of p<0.05 was used.

## Results

Enrichment of clozapine-treated patients within schizophrenia cases referred for immunology assessment and requiring immunoglobulin replacement therapy.

During the evaluation period, 1791 adults were assessed at ICW; 23 had a diagnosis of schizophrenia or schizoaffective disorders. We hypothesised that if clozapine-associated hypogammaglobulinaemia was clinically relevant, this would manifest in referral patterns. The mean clozapine prescription (online supplementary table 1) rate reported by the 2014 UK National Audit of Schizophrenia was 30%.^17^ We therefore expected a ratio of 7 clozapine:16 non-clozapine users. In contrast, we observed 17 patients with a history of clozapine use, corresponding to an OR of 6.48 (95% CI: 1.79 to 23.5), p=0.0072. This remained significant for prevalence estimates of clozapine use among the Welsh schizophrenia population up to 43% (online supplementary file S2). Patients receiving clozapine accounted for 6/7 (86%) of schizophrenia cases requiring immunoglobulin replacement therapy (IgRT), approximately 3% of our adult IgRT cohort. This compares with the international schizophrenia prevalence of 0.4%–1%.^18 19^ This suggests enrichment of clozapine-treated patients within our immunodeficiency cohort relative to the general population.

10.1136/jclinpath-2019-206235.supp2 Supplementary data

**Figure 1:**
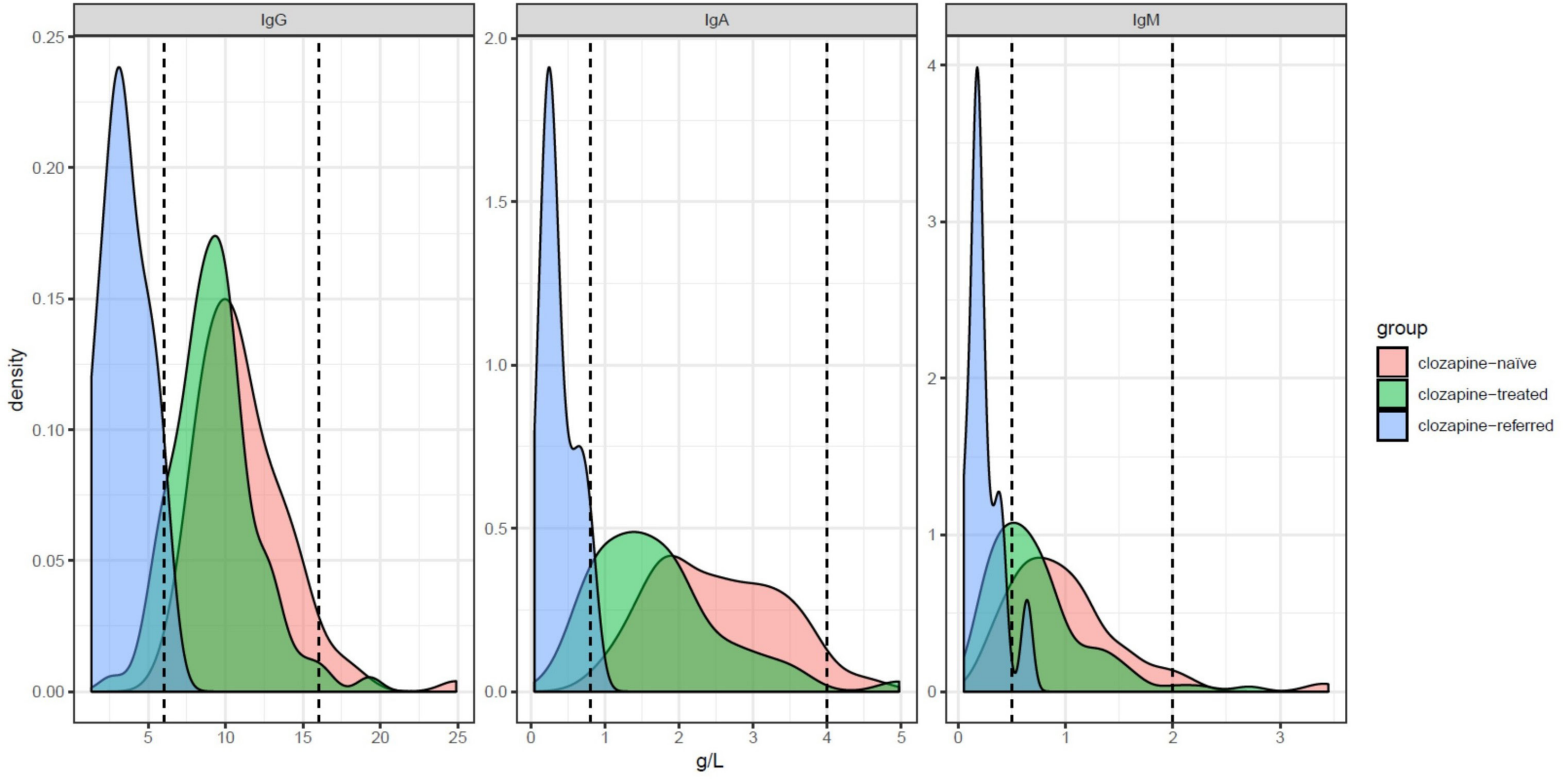
Immunoglobulin distribution in referred relative to referral cohort populations. Density plot showing distribution of serum immunoglobulin levels in patients receiving clozapine referred for immunology assessment (light blue, n=13 following removal of 4 patients, n=2 due to haematological malignancy and n=2 previously included within the case-control study). Comparison is made relative to the normal laboratory range (dotted lines, indicating 5th–95th percentiles) with comparison to immunoglobulin distributions in disease-control patient populations receiving clozapine (green, n=94) or alternative antipsychotic agents (pink, n=98) and following exclusion of individuals with alternative causes of hypogammaglobulinaemia.^12^

We next explored indication for referral and immunological finding at first assessment (individual patient details summarised—online supplementary file S1). Two clozapine-treatment patient with hypogammaglobulinaemia have been previously identified and reported.^12^ Recurrent infection was documented in 10/17 subjects (59%), predominately reflecting sinopulmonary infections. Four patients (24%) were referred with serum antibody levels below the fifth percentile without any antibiotic use in the preceding 12 months (summarised—table 1). A low calculated globulin (CG) (<23 g/L) was present in 15/17 (88%) of subjects receiving clozapine and was associated with reductions in IgG, IgA and IgM below the fifth percentiles in 14/17 subjects. We also found referral rates increased following the national introduction of CG screening to Wales during 2014, and prior to release of our initial report^12^ (online supplementary file S3). Consistent with CG reduction, serum immunoglobulin levels were also reduced (summarised in figure 1). Taken together, this is consistent with a clozapine-specific association with dysgammaglobulinaemia, and the utility of CG screening to stratify patients for specialist immunology assessment.

10.1136/jclinpath-2019-206235.supp3 Supplementary data

**Table 1:** Immunology assessment summary at initial clinical visit

	Schizophrenia with history of clozapine use (median, range)	Adult reference range (5th–95th percentile unless indicated*)
Number	17	
Age, years (median, range)	50 (36–63)	
Gender (M:F)	11:6	
Neutrophils (median, range)	4.3 (2.0–10.2)	1.7–7.5×10^9^/ L
Lymphocytes (median, range)	1.3 (0.8–2.1)	1.0–4.5×10^9^/L
IgG	3.20 (1.20–6.65)	6.00–16.0 g/L
IgA	0.26 (0.05–0.81)	0.80–4.00 g/L
IgM	0.17 (0.05–0.64)	0.50–2.00 g/L
CD3+ T cells	930 (380–1960)	800–2500×10^6^/ L
CD3+CD4+ T helper cells	600 (110–1350)	400–1500×10^6^/ L
CD3+CD8+ cytotoxic T cells	230 (60–560)	200–1100×10^6^/ L
CD19+ B cells	260 (100–450)	50–500×10^6^/ L
CD56+ natural killer cells	90 (20–460)	80–650×10^6^/ L
Naïve B cells (%B) (n=14)	83.8 (23.6–95.8)	58%–79.6%*
Marginal zone-like B cells (%B) (n=14)	11.0 (3.1–71.2)	7.4%–21.4%*
Class-switched memory B cells (%B) (n=16)	1.6 (0.2–11.6)	7.2%–18.9%*
Plasmablasts (%B) (n=6)	0.01 (0–0.04)	0.4–3.6*

Reference ranges for immunoglobulins^47^and lymphocyte subsets^48^ represent 5th–95th percentiles.

*B cell adult reference ranges represent interquartile range (adapted from Morbach *et al* 49). See also online supplementary table 1 for individual values.

See online supplementary file S1 for individual patient values.

### B-cell dysregulation associated with long-term clozapine use resembles CVID

**Figure 2:**
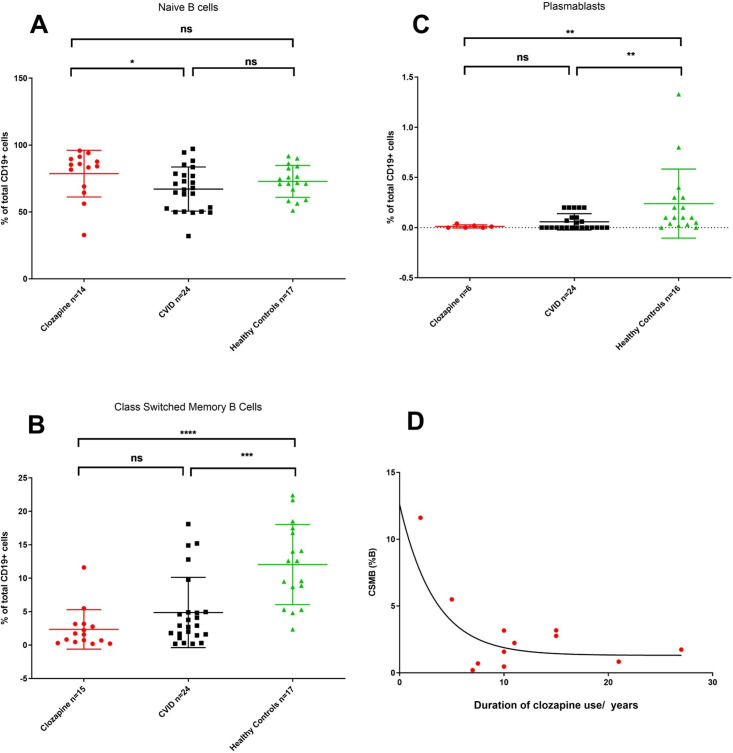
B-cell subsets in patients with schizophrenia with history of clozapine therapy (numbers as shown), common variable immunodeficiency (CVID, n=26) and healthy controls (n=17). B-cell subsets gated on CD19+ cells and defined as follows: (A) naïve B cells (CD27−IgD+IgM+); (B) class-switched memory B cells (CSMB, CD27+IgD−IgM−); (C) plasmablasts (CD19+CD27^hi^IgD-);. Non-parametric Mann-Whitney U test performed for non-normally distributed data, *p<0.05, **p<0.01, ***p<0.001, ****p<0.0001; (D) relationship of clozapine therapy (data available for n=12) and CSMB (%). Line of best fit shown represents single-phase exponential decay modelling for this relationship, r^2^=0.649. ns, not significant.

Clozapine users are closely monitored for side effects of neutropaenia and agranulocytosis, and all individuals showed normal neutrophil counts at first assessment. Lymphopaenia and reduction in T cells were present but largely confined to two individuals subsequently diagnosed with haematological malignancies (table 1; online supplementary file S2). Because the mechanisms underlying clozapine-associated hypogammaglobulinaemia remain unknown, we focused on B-cell clinical immunophenotyping data performed in isolation or as part of a full EUROClass evaluation.^16^ We took advantage of existing laboratory data to identify age-matched individuals with CVID (n=26) and healthy controls (n=16) to clozapine-treated patients (following exclusion of cases with haematological malignancy, n=15). Clozapine-treated individuals showed normal total and naive B-cell counts (Figure 2A) but reduction of class-switched memory B cells (CSMB) relative to healthy individuals (Mann-Whitney U test, p<0.0001), with the exception of a single patient who had discontinued clozapine after a 2-month trial and had normal immunoglobulin levels (figure 2B; online supplementary file S2). Immunophenotyping of plasmablasts was available for six individuals receiving clozapine and was comparable to CVID (Figure 2C), but significantly reduced compared with age-matched healthy controls (Mann-Whitney U test, p=0.004). We observed a general decline in CSMB with increasing duration of clozapine therapy (figure 2D), which appears independent of age (online supplementary file S4).

Vaccine-specific IgG responses are routinely evaluated as part of clinical assessment.^20^ At initial assessment, levels below putative protective thresholds were common with IgG to *H. influenzae* B (HiB) <1 μg/mL^21^ in 12/16 assessed (75%); *Pneumococc*al-IgG <50 mg/L^22^ in 15/16 (94%) and tetanus-IgG <0.1 IU/mL in 6/16 (38%) individuals tested. Post-*Menitorix* (HiB/MenC conjugated to tetanus toxoid) vaccination serology was assessed after 4 weeks, with 5/11 (45%) individuals failing to mount a HiB-IgG response ≥1 μg/mL, and 1/12 failing to exceed the ≥0.10 IU/mL postvaccination tetanus-IgG level defined by WHO.^23^ Following *Pneumovax II,* 6/16 (38%) individuals failed to develop an IgG response above a threshold of ≥50 mg/L^22^ (see online supplementary file S1 and online supplementary file S5 for vaccine response assessment).

### Reduction in infection burden following immunoglobulin replacement therapy

Our approach to antibody deficiency follows the previously reported findings.^15 20^ Cumulative follow-up for clozapine-associated hypogammaglobulinaemia now exceeds 61 patient-years (range 0.5–11 years), during which time 6/16 (38%) of clozapine-treated patients were commenced on IgRT. Over a 12-month period prior to replacement, these patients received a mean of 6.5 acute antibiotic courses (range 3–12, including one inpatient admission requiring parenteral therapy). Following IgRT, this fell to a median of 1.5 oral antibiotics courses per year (range 0–2 courses), with no infection-triggered hospital admissions. Replacement through IgG levels ranged from 5.5 to 9.6 g/L (mean 8.0 g/L).

### Partial recovery of immunoglobulin following clozapine discontinuation

**Figure 3:**
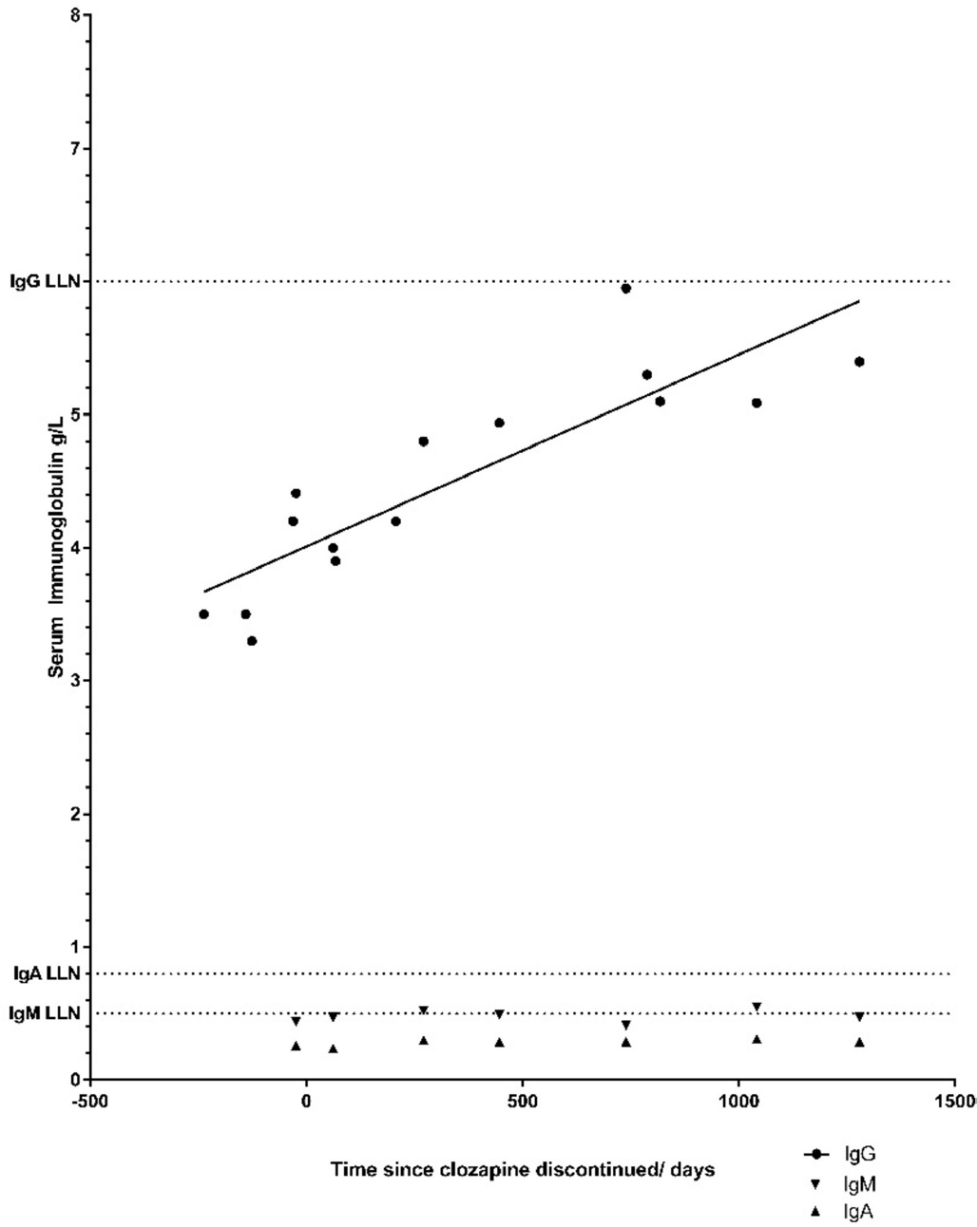
Gradual recovery of serum IgG postdiscontinuation of clozapine in a single patient without recourse to immunoglobulin replacement. LLN, lower limit of normal (see online supplementary S1, patient #7).

One patient (online supplementary table S1: IDs #7) discontinued clozapine due to the known side effect of neutropaenia, detected by the clozapine monitoring programme, providing a unique opportunity to examine reversibility of humoral dysregulation. In the absence of IgRT, patient #7 has demonstrated a gradual recovery in terms of serum IgG level from 3.5 to 5.95 g/L over 3 years, however IgA and IgM levels have remained depressed (figure 3). Patient #2 discontinued clozapine 2 years ago, having already commenced IgRT, with subsequent recovery of IgM from 0.22 to 0.86. IgA levels have remained below 0.24 g/L. Together, this provides support for reversibility in humoral function following clozapine-withdrawal, but suggests this process is gradual and limited. In contrast, clozapine cessation in patient #2 was associated with profound and acute relapse of psychiatric symptoms, requiring prolonged inpatient admission to a specialist mental health unit.

## Discussion

In this retrospective review of referrals to a national immunology centre, we found that individuals with schizophrenia receiving clozapine displayed clinically significant pan-hypogammaglobulinaemia, impaired vaccine responses and disturbed B-cell maturation with a proportion benefitting from IgRT. Consistent with a specific relationship to clozapine use, we observed enrichment of clozapine use among patients with schizophrenia undergoing immunology assessment or requiring IgRT relative to that reported in the wider population by a number of recent studies.^4 17 24^ The most common feature at referral was a low CG.^25^ Importantly, both clozapine and non-clozapine antipsychotic medications require routine liver function monitoring,^26^ arguing against a referral bias due to differential testing. While the use of CG screening is being developed in a number of countries, a lack of wider availability may limit the identification of antibody-deficient patients taking clozapine in other settings.^25 27 28^

Our finding of a temporal association between clozapine use and reduction in CSMB is consistent with our previous analysis of a separate cohort that hypogammaglobulinaemia appears a late manifestation of clozapine therapy.^12^ Our immunological findings associated with clozapine-associated hypogammaglobulinaemia is independently supported by a recently reported peripheral blood immune signature for patients with TRS revealing reduced plasmablasts and CSMB relative to disease and healthy controls.^29^ While this study focused on psychiatric status, the majority of such patients were clozapine-treated; immunoglobulin levels were not determined by this group.^29^ These findings also suggest that many clozapine-treated patients in our cohort and beyond may have received a diagnosis of CVID, according to internationally accepted criteria.^30^ We are conducting a wider survey of UK Immunology centres to examine this observation.

This study has several limitations, including its sample size and single-centre retrospective design. Prospective studies are required, for instance, to validate the association between therapy duration and effect on B-cell phenotypes, and to address causality. We speculate an effect targeting late-stage B-cell differentiation or survival may underlie the observed findings.^31^ However, at present, we cannot exclude a link to the underlying pathophysiology of schizophrenia, which like CVID remains largely unknown. Intriguingly, pathway analysis of schizophrenia associations have demonstrated marked enrichment at enhancers active in immune tissues, particularly B-lymphocyte lineages.^32^ Polypharmacy, multimorbidity and socioeconomic status are common and important factors complicating healthcare delivery in schizophrenia^33 34^ and might also contribute to the observed findings. Smoking is common in both clozapine-treated and clozapine-naïve patients with schizophreniapatients,^35^ predisposes to pneumonia^36^ and may lead to systemic steroid therapy for chronic obstructive pulmonary disorder. However, the reduced CSMB and pan-hypogammaglobulinaemia profiles we observed clearly differ from the patterns previously reported with both steroids or smoking.^37 38^ Other medication including metformin and antiepileptics have been described to have immunomodulatory effects in murine studies^39^ or case series^40^; however, do not fully explain the observed hypogammaglobulinaemia—in line with the rigorous exclusion criteria applied during our initial case-control study.^12^ The immunomodulatory effects of the dibenzodiazepine clozapine are increasingly recognised beyond agranulocytosis and neutropaenia.^41–43^ Here, we present evidence of gradual recovery of humoral immune function following clozapine withdrawal in the absence of IgRT.

From a practical standpoint, recovery of immunoglobulin levels appeared gradual and variable. Weighed against the florid relapse in psychiatric symptoms observed following clozapine discontinuation in one, we do not currently suggest discontinuation of clozapine in patients. This is in line with the unique efficacy of clozapine in TRS,^44^ and availability of treatment options to mitigate the risks associated with antibody deficiency. Finally, given evidence of increased risk of pneumonia within individuals with schizophrenia^7 45 46^, our finding of low levels of baseline immunity to common vaccinations within this patient group highlights a simple strategy for risk mitigation.

### Summary

We report the clinical features of clozapine-associated immunodeficiency identified following the introduction of CG screening in Wales and highlight clinically significant pan-hypogammaglobulinemia, impaired vaccine responses and new findings of disturbed B-cell maturation. In support of a drug-related effect, we show a temporal association between clozapine exposure and reduction in CSMB levels, and evidence of gradual reversibility following discontinuation. Clinicians should be alert for this diagnostic overlap with CVID. Patients may benefit from monitoring or clinical intervention in the form of vaccination, antibiotic prophylaxis or IgRT. Clozapine’s immunomodulatory effects are poorly understood, and further studies are required to delineate mechanism.

### Kudos plain language summary

The introduction of clozapine in the 1950s was a major therapeutic advance in the treatment of schizophrenia. It remains the gold standard therapy for approximately 3/10 individuals who fail to respond to initial management. Overall, clozapine improves symptoms and saves lives. Recent studies (2018) have suggested that clozapine therapy may be associated with a block in antibody production (causing antibody deficiency). This would predict patients receiving this therapy to be more likely to experience infections (such as pneumonia). This work examines patients referred to a major immunology centre since 2005, with focus on those receiving antipsychotic medicines (including clozapine). As predicted by the authors (but against the normal pattern of prescribing), clozapine was the most common antipsychotic used by patients referred with antibody deficiency. The authors go on to define the clinical and immunological features of this group, highlighting a close similarity to individuals without a known cause of antibody deficiency. Several clozapine-treated patients went on to receive antibody replacement therapy, successfully reducing their infection rate. By following these patients over time, they also saw that in one patient who stopped clozapine treatment this was associated with a gradual return in their major antibody (IgG). This adds support to the argument that clozapine therapy is associated with a drug-related (secondary) antibody deficiency. This is an important finding for immunologists around the work, who could easily confuse a drug-related cause of antibody deficiency with an inherited (primary) case. The authors remain cautious and suggest further studies are needed with greater size and following patients before and after they start/stop clozapine. This is because the disease processes underlying both schizophrenia and primary antibody deficiency remain largely unknown: meaning there could be important shared mechanisms linking both conditions.

**Take home messages**

- Clozapine remains the only effective medication for treatment-resistant schizophrenia (TRS); however, it is associated with an increased risk of pneumonia and death.
- Hypogammaglobulinaemia associated with patients with TRS receiving clozapine can result in a serious and significant infection burden, with patients often requiring intervention in the form of antibiotic prophylaxis and/or immunoglobulin replacement therapy.
- Hypogammaglobulinaemia appears to be at least partially reversible on clozapine cessation.
- Marked reduction in class-switched memory B cells is observed in patients with clozapine-associated hypogammaglobulinaemia, suggesting these individuals may currently be labelled as having primary antibody deficiency such as common variable immunodeficiency.

## References

[1] Owen MJ, Sawa A, Mortensen PB Schizophrenia. The Lancet 2016;388:86–97. 10.1016/S0140-6736(15)01121-6 PMC4940219 26777917

[2] Hor K, Taylor M Suicide and schizophrenia: a systematic review of rates and risk factors. J Psychopharmacol 2010;24:81–90. 10.1177/1359786810385490 20923923 PMC2951591

[3] Andrew A, Knapp M, McCrone P, et al Effective interventions in schizophrenia the economic case: a report prepared for the schizophrenia Commission. London: London School of Economics and Political Science, 2012.

[4] Cooper SCM, Etherington AM, Jayakumar S Report of the National Audit of Schizophrenia. Royal College of Psychiatrists, 2012.

[5] Leung JG, Hasassri ME, Barreto JN, et al Characterization of admission types in medically hospitalized patients prescribed clozapine. Psychosomatics 2017;58:164–72. 10.1016/j.psym.2016.11.013 28153339

[6] Stoecker ZR, George WT, O'Brien JB, et al Clozapine usage increases the incidence of pneumonia compared with risperidone and the general population: a retrospective comparison of clozapine, risperidone, and the general population in a single hospital over 25 months. Int Clin Psychopharmacol 2017;32:155–60. 10.1097/YIC.0000000000000162 28059928

[7] Kuo C-J, Yang S-Y, Liao Y-T, et al Second-Generation antipsychotic medications and risk of pneumonia in schizophrenia. Schizophr Bull 2013;39:648–57. 10.1093/schbul/sbr202 22282455 PMC3627761

[8] Rohde C, Polcwiartek C, Kragholm K, et al Adverse cardiac events in out-patients initiating clozapine treatment: a nationwide register-based study. Acta Psychiatr Scand 2018;137:47–53. 10.1111/acps.12827 29064084

[9] Taylor DM, Douglas-Hall P, Olofinjana B, et al Reasons for discontinuing clozapine: matched, case-control comparison with risperidone long-acting injection. Br J Psychiatry 2009;194:165–7. 10.1192/bjp.bp.108.051979 19182180

[10] Mustafa FA, Burke JG, Abukmeil SS, et al "Schizophrenia past clozapine": reasons for clozapine discontinuation, mortality, and alternative antipsychotic prescribing. Pharmacopsychiatry 2015;48:11–14. 10.1055/s-0034-1394397 25376977

[11] Dyer C Coroners warn health Secretary of clozapine deaths. BMJ 2018;363:k5421 10.1136/bmj.k5421 30591547

[12] Ponsford M, Castle D, Tahir T, et al Clozapine is associated with secondary antibody deficiency. Br J Psychiatry 2018:1–7. 10.1192/bjp.2018.152 PMC6429246 30259827

[13] Ponsford MJ, Jolles S Antibody deficiency in patients taking clozapine. BMJ 2019;48:l483 10.1136/bmj.l483 30718272

[14] van Vollenhoven RF, Emery P, Bingham CO, et al Long-Term safety of rituximab in rheumatoid arthritis: 9.5-year follow-up of the global clinical trial programme with a focus on adverse events of interest in RA patients. Ann Rheum Dis 2013;72:1496–502. 10.1136/annrheumdis-2012-201956 23136242 PMC3756452

[15] Patel SY, Carbone J, Jolles S The expanding field of secondary antibody deficiency: causes, diagnosis, and management. Front Immunol 2019;10 10.3389/fimmu.2019.00033 PMC6376447 30800120

[16] Wehr C, Kivioja T, Schmitt C, et al The EUROclass trial: defining subgroups in common variable immunodeficiency. Blood 2008;111:77–85. 10.1182/blood-2007-06-091744 17898316

[17] Cooper S, Crawford M, Dicks S Report of the second round of the National Audit of Schizophrenia (NAS2). Royal College of Psychiatrists, 2014 https://www.rcpsych.ac.uk/improving-care/ccqi/national-clinical-audits/national-clinical-audit-of-psychosis/national-audit-schizophrenia

[18] Saha S, Chant D, Welham J, et al A systematic review of the prevalence of schizophrenia. PLoS Med 2005;2:e141 10.1371/journal.pmed.0020141 15916472 PMC1140952

[19] Bhugra D The global prevalence of schizophrenia. PLoS Med 2005;2:e151–75. 10.1371/journal.pmed.0020151 15916460 PMC1140960

[20] Jolles S, Chapel H, Litzman J When to initiate immunoglobulin replacement therapy (IGRT) in antibody deficiency: a practical approach. Clin Exp Immunol 2017;188:333–41. 10.1111/cei.12915 28000208 PMC5422851

[21] Käyhty H, Peltola H, Karanko V, et al The protective level of serum antibodies to the capsular polysaccharide of Haemophilus influenzae type B. J Infect Dis 1983;147:1100 10.1093/infdis/147.6.1100 6602191

[22] Chua I, Lagos M, Charalambous BM, et al Pathogen-Specific IgG antibody levels in immunodeficient patients receiving immunoglobulin replacement do not provide additional benefit to therapeutic management over total serum IgG. J Allergy Clin Immunol 2011;127:1410–1. 10.1016/j.jaci.2011.01.035 21376379

[23] Colombet I, Saguez C, Sanson-Le Pors M-J, et al Diagnosis of tetanus immunization status: multicenter assessment of a rapid biological test. Clin Diagn Lab Immunol 2005;12:1057–62. 10.1128/CDLI.12.9.1057-1062.2005 16148171 PMC1235798

[24] Tungaraza TE, Ahmed W, Chira C, et al Prescribing pattern of clozapine and other antipsychotics for patients with first-episode psychosis: a cross-sectional survey of early intervention teams. Ther Adv Psychopharmacol 2017;7:103–11. 10.1177/2045125316683151 28348730 PMC5354130

[25] Holding S, Khan S, Sewell WAC, et al Using calculated globulin fraction to reduce diagnostic delay in primary and secondary hypogammaglobulinaemias: results of a demonstration project. Ann Clin Biochem 2015;52:319–26. 10.1177/0004563214545791 25024432

[26] Taylor DM, Paton C, Kapur S The Maudsley prescribing guidelines. Informa Healthcare, 2009.

[27] Jolles S, Borrell R, Zouwail S, et al Calculated globulin (CG) as a screening test for antibody deficiency. Clin Exp Immunol 2014;177:671–8. 10.1111/cei.12369 24784320 PMC4137851

[28] Pecoraro A, Jolles S, Crescenzi L, et al Validation of calculated globulin (CG) as a screening test for antibody deficiency in an Italian university hospital. Curr Pharm Biotechnol 2018;19:728–33. 10.2174/1389201019666180808163311 30091407

[29] Fernandez-Egea E, Vértes PE, Flint SM, et al Peripheral immune cell populations associated with cognitive deficits and negative symptoms of treatment-resistant schizophrenia. PLoS One 2016;11:e0155631–e. 10.1371/journal.pone.0155631 27244229 PMC4887013

[30] Bonilla FA, Barlan I, Chapel H, et al International consensus document (icon): common variable immunodeficiency disorders. J Allergy Clin Immunol 2016;4:38–59. 10.1016/j.jaip.2015.07.025 PMC4869529 26563668

[31] Ponsford MJ, Pecoraro A, Jolles S Clozapine-Associated secondary antibody deficiency. Curr Opin Allergy Clin Immunol 2019;19:553–62. 10.1097/ACI.0000000000000592 31567398

[32] Schizophrenia Working Group of the Psychiatric Genomics Consortium Biological insights from 108 schizophrenia-associated genetic loci 2014;511:421–7. 10.1038/nature13595 PMC4112379 25056061

[33] Ballon J, Stroup TS Polypharmacy for schizophrenia. Curr Opin Psychiatry 2013;26:208–13. 10.1097/YCO.0b013e32835d9efb 23318662 PMC4026924

[34] Legge SE, Hamshere M, Hayes RD, et al Reasons for discontinuing clozapine: a cohort study of patients commencing treatment. Schizophr Res 2016;174:113–9. 10.1016/j.schres.2016.05.002 27211516 PMC5756540

[35] Kelly C, McCreadie RG, Habits S Smoking habits, current symptoms, and premorbid characteristics of schizophrenic patients in Nithsdale, Scotland. Am J Psychiatry 1999;156:1751–7. 10.1176/ajp.156.11.1751 10553739

[36] Bello S, Menéndez R, Antoni T, et al Tobacco smoking increases the risk for death from pneumococcal pneumonia. Chest 2014;146:1029–37. 10.1378/chest.13-2853 24811098

[37] Brandsma C-A, Hylkema MN, Geerlings M, et al Increased levels of (class switched) memory B cells in peripheral blood of current smokers. Respir Res 2009;10:108 10.1186/1465-9921-10-108 19909533 PMC2779187

[38] Wirsum C, Glaser C, Gutenberger S, et al Secondary antibody deficiency in glucocorticoid therapy clearly differs from primary antibody deficiency. J Clin Immunol 2016;36:406–12. 10.1007/s10875-016-0264-7 26980224

[39] Ursini F, Russo E, Pellino G, et al Metformin and Autoimmunity: A "New Deal" of an Old Drug. Front Immunol 2018;9:1236 10.3389/fimmu.2018.01236 29915588 PMC5994909

[40] Eom T-H, Lee H-S, Jang P-S, et al Valproate-Induced panhypogammaglobulinemia. Neurol Sci 2013;34:1003–4. 10.1007/s10072-012-1153-3 22797722

[41] Idänpään-Heikkilä J, Alhava E, Olkinuora M, et al Clozapine and agranulocytosis. The Lancet 1975;306:611 10.1016/S0140-6736(75)90206-8 51442

[42] Røge R, Møller BK, Andersen CR, et al Immunomodulatory effects of clozapine and their clinical implications: what have we learned so far? Schizophr Res 2012;140:204–13. 10.1016/j.schres.2012.06.020 22831769

[43] Green LK, Zareie P, Templeton N, et al Enhanced disease reduction using clozapine, an atypical antipsychotic agent, and glatiramer acetate combination therapy in experimental autoimmune encephalomyelitis. Mult Scler J Exp Transl Clin 2017;3:205521731769872 10.1177/2055217317698724 PMC5453410 28607752

[44] NICE Psychosis and schizophrenia in adults: prevention and management. Available: https://www.nice.org.uk/guidance/cg1782014 32207892

[45] Seminog OO, Goldacre MJ Risk of pneumonia and pneumococcal disease in people with severe mental illness: English record linkage studies. Thorax 2013;68:171–6. 10.1136/thoraxjnl-2012-202480 23242947

[46] Haddad PM Current use of second-generation antipsychotics may increase risk of pneumonia in people with schizophrenia. Evid Based Ment Health 2013;16:109 10.1136/eb-2013-101441 24091617

[47] Rowbottom A, Wild GD Pru Handbook of clinical immunology. 9th edn, 2004: 103–4.

[48] Comans-Bitter WM, de Groot R, van den Beemd R, et al Immunophenotyping of blood lymphocytes in childhoodReference values for lymphocyte subpopulations. J Pediatr 1997;130:388–93. 10.1016/S0022-3476(97)70200-2 9063413

[49] Morbach H, Eichhorn EM, Liese JG, et al Reference values for B cell subpopulations from infancy to adulthood. Clin Exp Immunol 2010;162:271–9. 10.1111/j.1365-2249.2010.04206.x 20854328 PMC2996594

