# Supplementary material for: Clinical and laboratory characteristics of clozapine-treated patients with schizophrenia referred to a national immunodeficiency clinic reveals a B-cell signature resembling common variable immunodeficiency (CVID)
Source: J Clin Pathol. 2020 Feb 24;73(9):587–92. doi: 10.1136/jclinpath-2019-206235 (PMC7476264; doi:10.1136/jclinpath-2019-206235)
Supplement: Supplementary data [file jclinpath-2019-206235supp002.pdf]

Supplementary S2: *The clinical and laboratory characteristics of clozapine treated schizophrenia patients in immunodeficiency clinic*

Supplementary Table 2

Fisher's exact test performed to assess referral patterns of clozapine vs non-clozapine treated schizophrenia patients referred for Immunology Assessment at ICW. Different tables correspond to increasing estimates for clozapine use prevalence amongst the Welsh schizophrenia population.

Contingency of Under regular review - 30% prevalence clozapine use

Table Analyzed	Under regular review - 30%		
Fisher's exact test			
P value	0.0072		
P value summary	**		
One- or two-tailed	Two-tailed		
Statistically significant? (alpha<0.05)	Yes		
Strength of association			
Odds ratio	6.48		
95% confidence interval	1.79 to 23.5		
Data analyzed	Clozapine	No Clozapine	Total
Observed	17	6	23
Expected	7	16	23
Total	24	22	46

Contingency of Under regular review - 33% prevalence clozapine use

Table Analyzed	Under regular review - 33%		
Fisher's exact test			
P value	0.0169		
P value summary	*		
One- or two-tailed	Two-tailed		
Statistically significant? (alpha<0.05)	Yes		
Strength of association			
Odds ratio	5.31		
95% confidence interval	1.50 to 18.8		
Data analyzed	Clozapine	No Clozapine	Total
Observed	17	6	23
Expected	8	15	23
Total	25	21	46

Supplementary S2: *The clinical and laboratory characteristics of clozapine treated schizophrenia patients in immunodeficiency clinic*

Contingency of Under regular review - 39% prevalence clozapine use

Table Analyzed	Under regular review - 39%		
Fisher's exact test			
P value	0.0361		
P value summary	*		
One- or two-tailed	Two-tailed		
Statistically significant? (alpha<0.05)	Yes		
Strength of association			
Odds ratio	4.41		
95% confidence interval	1.26 to 15.4		
Data analyzed	Clozapine	No Clozapine	Total
Observed	17	6	23
Expected	9	14	23
Total	26	20	46

Contingency of Under regular review - 43% prevalence clozapine use

Table Analyzed	Under regular review - 43%		
Fisher's exact test			
P value	0.0712		
P value summary	ns		
One- or two-tailed	Two-tailed		
Statistically significant? (alpha<0.05)	No		
Strength of association			
Odds ratio	3.68		
95% confidence interval	1.06 to 12.8		
Data analyzed	Clozapine	No Clozapine	Total
Observed	17	6	23
Expected	10	13	23
Total	27	19	46
