# Supplementary material for: Clinical and laboratory characteristics of clozapine-treated patients with schizophrenia referred to a national immunodeficiency clinic reveals a B-cell signature resembling common variable immunodeficiency (CVID)
Source: J Clin Pathol. 2020 Feb 24;73(9):587–92. doi: 10.1136/jclinpath-2019-206235 (PMC7476264; doi:10.1136/jclinpath-2019-206235)

Supplementary Figure, S3: Vaccine specific-IgG response assessment

See text and supplementary S1 for details.

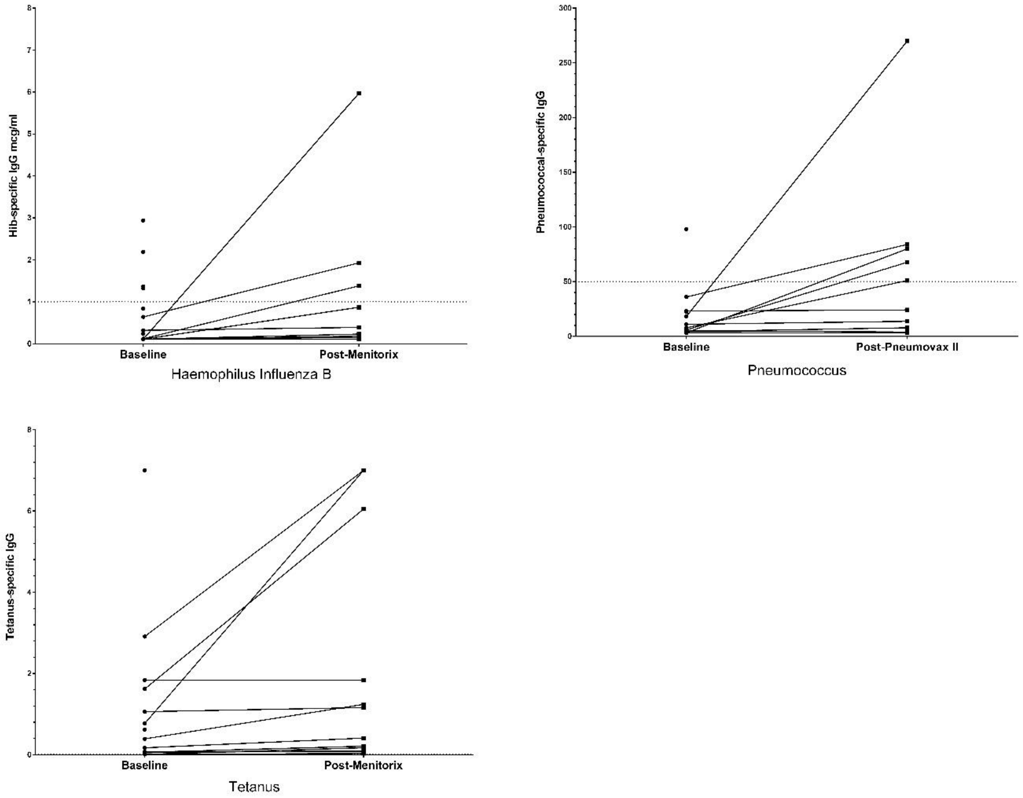

Supplementary S4: Decline in class-switched memory B-cell populations in individuals receiving clozapine is not readily explained by ageing.

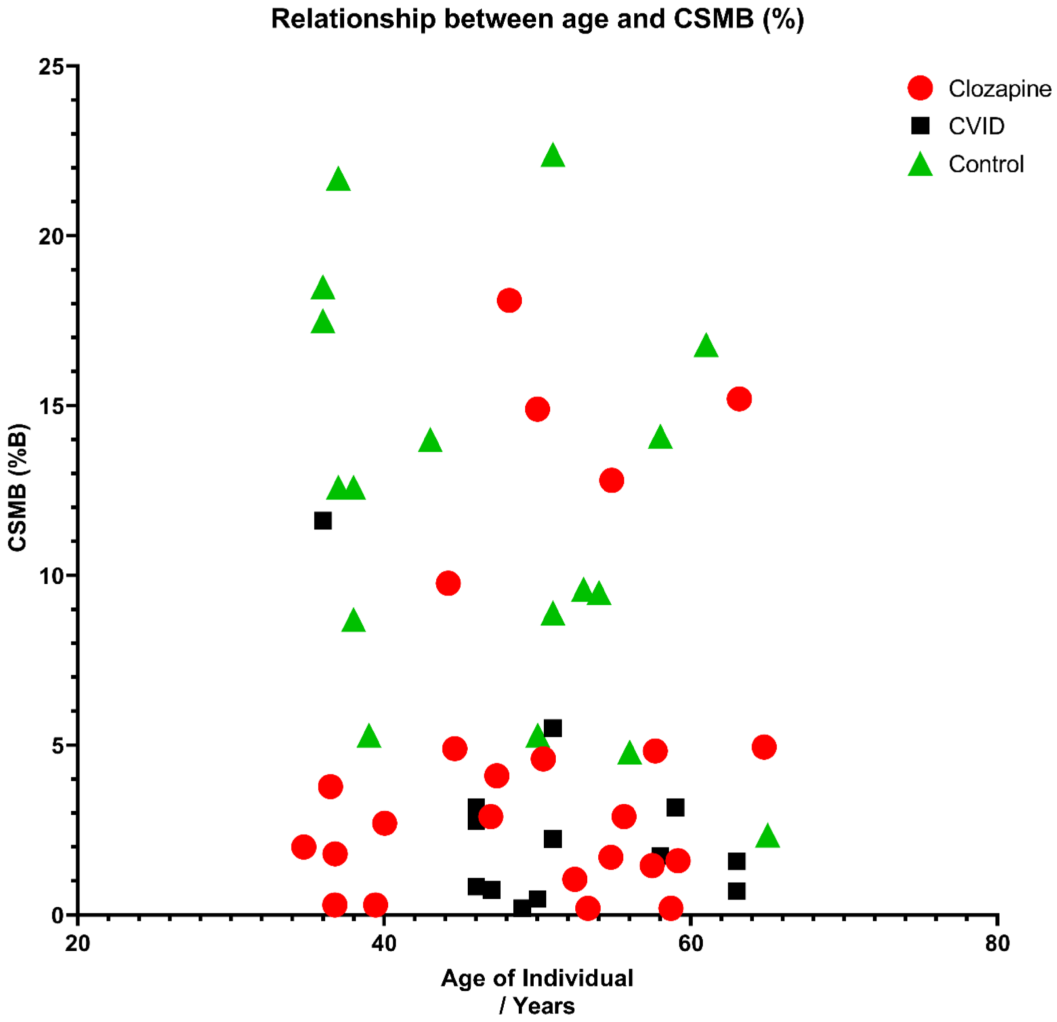

Supplementary, S5: Cumulative Referrals to ICW with a diagnosis of schizophrenia in relation to introduction of calculated globulin screening.

The national calculated globulin screening programme was setup during 2014, with roll-out complete across all health boards by November 2014. Consequently, any adult liver function test with low calculated globulin triggers the laboratory comment "*Low calculated globulin- may represent antibody deficiency. Consider immunoglobulins if there is a history of infections*".

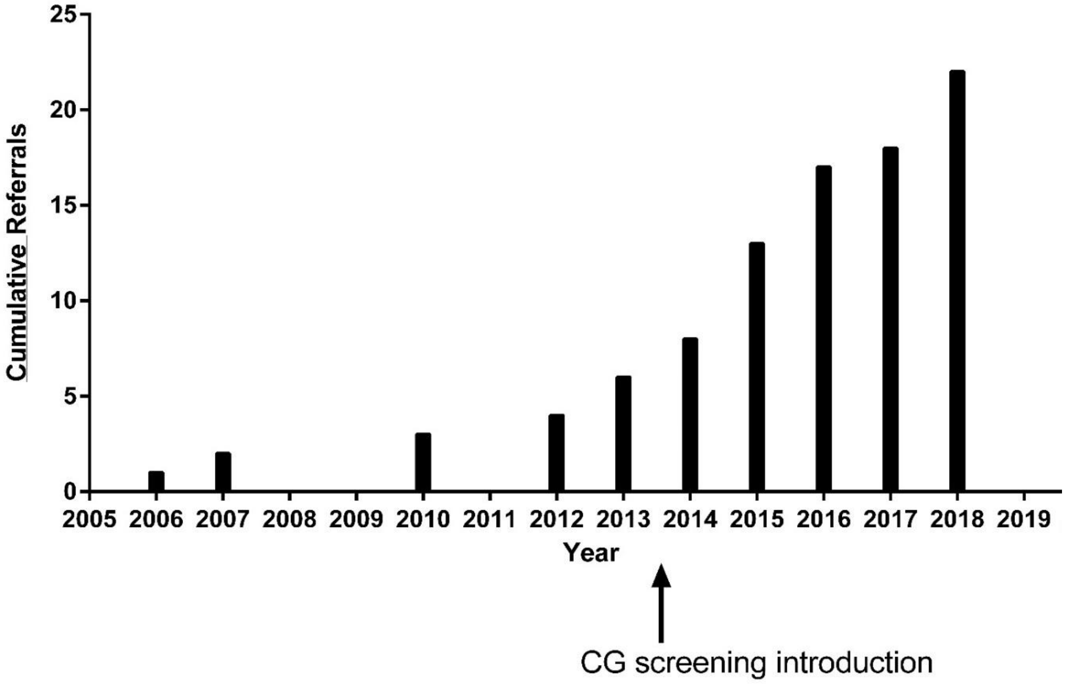

Supplement: Supplementary data [file jclinpath-2019-206235supp003.pdf]
